# Small Cell (Neuroendocrine) Carcinoma of the Cervix: An Analysis for 19 Cases and Literature Review

**DOI:** 10.3389/fcimb.2022.916506

**Published:** 2022-07-13

**Authors:** JunLing Lu, Ya Li, Jun Wang

**Affiliations:** Department of Gynecology and Obstetrics, The Second Affiliated Hospital of Dalian Medical University, Dalian, China

**Keywords:** neuroendocrine carcinoma, small cell, human papillomavirus, diagnosis, therapy

## Abstract

Cervical SCNEC is a rare and highly malignant invasive tumor. The incidence is low, at less than 5% of all cervical cancers. Moreover, most patients with small cell carcinoma are interrelated with high risk HPV (more familiar HPV 18). Compared to squamous cell carcinoma or adenocarcinoma, patients of cevical SCNEC are more prone to lymph node invasion early, so the clinical manifestation is usually local or distant metastasis. We summarized the clinical features of 19 patients with cervical small cell carcinoma in the Second Affiliated Hospital of Dalian Medical University from 2012 to 2021, and retrospectively analyzed data from 1576 patients in 20 related studies and more than 50 pieces of literature in recent years by searching PubMed, Google schalor, Cochrane Library, Clinicalkey, and other databases. The collected patient data included age, clinical manifestation, TCT, HPV detection, the size and morphology of the tumor, local invasion depth, stage, lymph node status, initial treatment method, tumor-free survival, and so on. The positive rates of CGA, SYN, and CD56 in our cases were high, and NSE was a moderately sensitive index. P16 and Ki67 were the most sensitive, and all patients were positive. We found that multimodal treatment can indeed improve tumor-free survival (DFS), but the prognosis of patients is still very poor. For the early stages, our treatment principles refer to the guidelines of SGO, international gynecological cancer Cooperation (GCIG), and NCCN. We suggest a combination of surgery, radiotherapy, and chemotherapy. However, the general state of advanced patients is poor, whether they can tolerate the operation after neoadjuvant chemotherapy, whether the operation area can remain tumor-free, and whether this treatment will prolong the survival time of patients still need to be further discussed. In order to better prolong the tumor-free survival and prognosis of patients, we need to find gene changes suitable for targeted therapy, so as to complete the clinical application of these treatment methods. Further works are needed to explore more effective therapy for cervical SCNEC.

## Introduction

Cervical cancer is the second most prominentcause of cancer-related death among women worldwide (Siegel et al., 2014). With the advent of the vaccine era and the widespread application of cervical screening, the incidence rate has declined, but tends to skew younger. A neuroendocrine tumor is a seldom-seen disease of the female reproductive system, and the cervix is the most common primary location (Salvo et al., 2019). In 2014, the World Health Organization divided cervical neuroendocrine tumors into two categories, which were low level (formerly known as carcinoid and atypical carcinoid) and high level (formerly known as small cell cancer or large cell neuroendocrine cancer) (Kurman et al., 2014). Cervical small cell neuroendocrine carcinomas are highly invasive and progress rapidly, their incidence is less than 5% of all cervical cancers. About 80% cervical SCNEC concerned high risky HPV (more familiar with HPV 18). Compared to squamous cell carcinoma or adenocarcinoma, patients are more prone to lymph node invasion early, which usually shows as local and distant metastases in clinic (Park et al). By searching PubMed, Google scholar, Cochrane Library, clinicalkey, and other databases, this paper retrospectively analyzed data from 1576 patients in 20 related studies and more than 50 papers in recent years of small cell carcinoma of the cervix in recent years and combined with 19 cases of this disease treated in our hospital from 2012 to 2021. We summarize the disease characteristics, pathogenesis, and relationship with HPV infection, which is in order to explore the timely diagnosis before canceration and improve the treatment, so as to improve the prognosis and survival rate of patients.

## Methods

The clinical data of small cell cancer treated in our hospital from 2012 to 2021 were analyzed retrospectively. The criteria for selecting cases were patients with a pathological diagnosis of small cell neuroendocrine carcinoma. The collected patient data included age, clinical manifestation, TCT, HPV detection, the size and morphology of the tumor, local invasion depth, stage, lymph node status, lymphatic vascular space involvement (LVSI), initial treatment method, tumor-free survival, follow-up interval, and mortality. All information was obtained through access to medical records. The research was approved by the ethics committee of the Second Affiliated Hospital of Dalian Medical University.

## Results

We found 19 cases of cervical small cell carcinoma in our hospital between 2012 to 2021. The total incidence rate was 1.5%. **Table 1** shows the general statistics of 19 patients. The median age was 48 years (with a range from 26 years to 70 years) which is five years younger than that of worldwide cervical carcinoma (Arbyn et al., 2020). The main clinical manifestation was irregular vaginal bleeding. In total 52.63% (10/19) of patients had postcoital bleeding before irregular vaginal spotting. Also, 36.84% (7/19) of patients had aqueous secretion or persistent vaginitis before abnormal bleeding, while 10.52% (2/19) of patients had no obvious symptoms before irregular vaginal bleeding. Only 47.37% (9/19) had TCT and HPV tests, the others were directly diagnosed by biopsy due to gynecological examination of suspected cervical cancer. While 55.56% (5/9) of the total had abnormal results with TCT. Among the patients with abnormal results, one patient was ASCUS(Atypical squamous cells of unknown significance), three patients were AGC(Atypical glandular cells), and one patient was ASC-H(Atypical squamous cells, not excluding high-grade squamous intraepithelial lesion). Simultaneously, we calculated that the HPV infection rate was 77.77% (7/9). In our study, we found that HPV infection was mainly high-risk 18 HPV positive. Five cases were found to be infected with HPV-18,with one being infected with HPV-18 and HPV-58 at the same time. In addition, one person was HPV-16 positive and one person was HPV-52 positive. In our cases, the median disease-free survival (DFS) of patients accepting multiple combination therapy was 15.5 months. **Table 2** shows the stage distribution, recurrence, treatment modalities, and prognosis of our cases. Nine patients with stage IB accepted radical hysterectomy and pelvic lymphadenectomy. Because patients before 2018 did not have stage IB3, we reassessed them according to their case data. All patients received adjuvant therapy after their operation, including pelvic external irradiation (EBRT) and chemotherapy combined with Etoposide and Cisplatin monthly. Three patients with stage IIB received pelvic three-dimensional transparent radiotherapy combined with synchronous DDP low-dose chemotherapy. Two patients with stage IIIB were given combined chemotherapy (EP+COA). Five stage IV patients mainly received combined chemotherapy with Etoposide and Cisplatin and some symptomatic treatment. **Table 3** describes the evaluation of tumor characteristics in nine surgically treated patients in detail. Two patients (2/9) had coarse tumors with a diameter greater than or equal to 4cm at diagnosis, and seven (7/9) patients had deep interstitial infiltration (≥ 1/2 of the matrix). All patients had intravascular tumor thrombus. Only one patient found pelvic lymph node metastasis, but there were seven patients in stage IB of recurrence.

**Table 1:** Ordinary statistics of patients.

Variable	Figure	Literature data (52 articles,1576 patients)
Median Age, [range], years	48 (26-70)	the average age at diagnosis of worldwide cervical cancer was 53 years (40)
Irregular bleeding	100%	
postcoital bleeding before irregular vaginal bleeding	52.63% (10/19)	
abnormal results with TCT	55.56% (5/9)	
Median tumor-free survival(months)	15.5	Of the 179 eligible patients 104 were stage I, 19 stage IIA, 23 stage IIB, 9 stage III, 24 stage IV. The median tumor-free survival was16 months (Wang et al., 2012)
HPV infection rate	77.77% (7/9)	55% of cases were HPV16 positive; 41% were positive for HPV18 and 4% were positive for other types (Alejo et al., 2018) 15% of cases were HPV16 positive; 70% were positive for HPV18 and 15% were positive for other types (Stoler et al., 1991)
HPV-18	71.42% (5/7)	
HPV-16	14.29% (1/7)	
HPV-52	14.29% (1/7)	
Tumor metastasis site		
pelvic lymph node	6	
Lung	3	
Distant metastasis such as brain or bone	8 (brain3, bone5)	

(Distant metastasis was found by imaging examination).

**Table 2:** Treatment and prognosis of different stages.

Stage	Number	Number and interval of recurrence(average)	Treatment modalities
IB	IB1:3 IB2:4 IB3:2	IB1:2,36months IB2:3,28months IB3:2,18months	Radical hysterectomy and pelvic lymphadenectomy + EBRT and chemotherapy (Etoposide + Cisplatin) monthly.
IIB	3	3,8months	pelvic three-dimensional transparent radiotherapy combined with synchronous DDP low-dose chemotherapy.
IIIB	2	2,6 months	combined chemotherapy.
IV	5	5,3 months	combined chemotherapy with Etoposide and Cisplatin and some symptomatic treatment.
Total	19	17	

**Table 3:** Tumor features (Number=9).

Classification	Number
Tumor size >4 cm	2
Exogenous	7
Endogenous	2
Depth of invasion	
More than 1/2 cervical thickness	7
Vascular tumor thrombus	9
Pelvic LN(+)	2

**Table 4** describes details of the immunohistochemistry related to small cell carcinoma in our case. We can see that SYN, CD56, CGA, p16, and Ki67 are relatively sensitive.

**Table 4:** Immunohistochemistry.

	SYN	CD56	NSE	CGA	CK8/18	CK5/6	p16	Ki67	EMA	Vim	LCA	CK7	P40
positive	13(14)	11(13)	2(4)	11(14)	3(3)	2(5)	5(5)	8(8)	1(3)	1(4)	0(5)	2(3)	2(3)
negative	1(14)	3(13)	2(4)	3(14)	0(3)	3(5)	0(5)	0(8)	2(3)	3(4)	5(5)	1(3)	1(3)

## Discussion

### Cervical SCNEC and HPV

Cervical small cell neuroendocrine carcinoma (SCNEC) is a rare invasive cancer, accounting for no more than 3% of cervical cancer. Due to the high incidence of early lymph nodes and distant metastasis, it is usually found in the late stage (Chen et al., 2008; Cohen et al., 2010; Wharton et al., 2020). In our case, the proportion of cervical SCNEC is 1.5%, which is basically consistent with formerly reports. It is reported that the five year survival rate if diagnosed in the early stages is 30-46%, but only 0 – 15% at the advanced stage (Dongol et al., 2014). The early stage according to 2022 NCCN Guidelines Version 1 refers to stages IA1-IB2 and IIA1, while the advanced stage includes stages IB3, IIA2, and IIB-IV respectively (Abu-rustum et al., 2022). Moreover, a study has shown that despite the increase in comprehensive therapy, the prognosis of advanced patients remains worse (Lorusso et al., 2014). In our cases, small cell carcinoma patients have the symptoms of irregular vaginal bleeding. Before vaginal bleeding, 52.63% of patients experienced post-sexual bleeding. Among the patients screened for TCT and HPV, 55.56% have abnormal TCT and 77.77% have an HPV infection. This once again confirms the importance of cervical precancerous screening. Numerous clinical studies have shown that HPV infection is the main cause of cervical cancer (Small et al., 2017), and some studies have also confirmed that HPV infection is closely related to cervical SCNEC (Atienza-Amores et al., 2014; Dores et al., 2015). In the study by Alejo, 86% of neuroendocrine tumors were infected with HPV, mainly subtypes 16 and 18, and the positive rate of other subtypes was only 4%. Their data confirmed the correlation between cervical SCNEC, HPV, and p16^INK4a^ overexpression (Alejo et al., 2018). Peifeng Li et al. retrospectively analyzed the clinical data of 30 cases in the primary period of cervical SCNEC. They concluded that infection with multiple HPV subtypes was not associated with disease severity or prognosis (Li et al., 2018). Xuan Pei et al. detected seven high-risk HPV types by quantitative multiplex PCR. They found that cervical SCNEC was particularly related to HPV 18 subtypes. The genetic changes of patients were caused by high risk HPV and mutations in frequent neuroendocrine cancers (Pei et al., 2021). Moreover, several studies have also demonstrated that HPV18 was specifically related to cervical SCNEC (Stoler et al., 1991; Ishida et al., 2004). In our cases, patients were mainly infected with HPV 18 (71.42%). This is consistent with the results of many studies. How to detect and block the occurrence of small cell carcinoma in time before its occurrence is the main difficulty we must overcome in the future.

### Immunohistochemistry and Gene Detection

It is reported that commonly used neuroendocrine staining agents are chromogranin A (CGA), synaptophysin (SYN), CD56, and neuron-specific enolase (NSE). However, cervical SCNEC has not always responded to the neuroendocrine staining agents including CGA, SYN, and NSE (Stoler et al., 1991; Abeler et al., 1994; Conner et al., 2002). In our cases, the positive rates of the above indicators were 78.57% (CGA), 92.86% (SYN), 84.62% (CD56), and 50% (NSE). The sensitivity of p16 and Ki67 was high, and the patients were all positive. Inzani et al. found that 87.5% of patients had high expression of p16 protein, and more than half of the patients expressed SST5, SST2, and CDX2, while P63 and P40 were negative (Inzani et al., 2020). Changes in gene sequences are very important for the occurrence and progression of tumors. Deyin Xing et al. demonstrated the recurring gene changes referring to MAPK, PI3K/Akt/mTOR, and p53/BRCA pathways in cervical SCNEC by using target next-generation gene sequencing technology (Xing et al., 2018). In the future, the existence of alterations in gene sequences may be used for targeted therapy.

### Therapy of Cervical SCNEC

Due to the features of cervical SCNEC being low morbidity, highly malignant, powerful invasiveness, and poor prognosis, there is no accordant standard therapeutic regime now. In addition, given the insufficiency of prospective studies on therapeutic effects, the results of retrospective research are inconsistent, and the treatment methods are still controversial. In light of its special pathology, the therapeutic schedule can consult the methods of cervical cancers or other neuroendocrine cancers, such as small-cell carcinoma of the lung (SCLC), Merkel cell carcinoma (MCC), gastrointestinal neuroendocrine cancer, and so on (Becker et al., 2017; Sabari et al., 2017; Coggshall et al., 2018). Some studies have shown that the main treatment methods for cervical SCNEC include surgery, chemotherapy, and radiotherapy (Bermudez et al., 2001; Cohen et al., 2010; Gadducci et al., 2017). Some scholars demonstrated that the overall survival rate of patients with primary radical surgery was better than those who received direct radiotherapy. They preferred radical surgery combined with chemotherapy for the early stage. They found that patients receiving adjuvant chemotherapy had improved disease-free survival and reduced pelvic recurrence, and the overall survival rate tended to improve (Ishikawa et al., 2018). But Wang et al. indicated that the progression-free survival rate of radical surgery was worse (Wang et al., 2012). In our case, nine patients with stage IB accepted radical hysterectomy, and five of them received combined treatment after operation. Although seven of them recurred, we found a longer recurrence interval in patients who received early surgery combined with radiotherapy and chemotherapy. We summarized the case characteristics of non-recurrent patients and found that they all received satisfactory radical surgery and comprehensive treatment with radiotherapy and chemotherapy. Therefore, we still tend to recommend systemic treatment of early patients with surgery combined with concurrent radiotherapy and chemotherapy. For stages IA1-IB2 and IIA1, our treatment principles refer to the guidelines of SGO, international gynecological cancer Cooperation (GCIG), and NCCN, which all suggested radical surgery and adjuvant chemotherapy (Gardner et al., 2011; Stecklein et al., 2016). In our case, we often choose radiotherapy and chemotherapy to cure advanced-stage or recurrent patients. Caruso et al. retrospectively analyzed the patients who underwent a complete operation after neoadjuvant chemotherapy (NACT) in the later period. They found that compared with radiotherapy and chemotherapy, the surgical treatment may produce similar results. The possible benefit is to retain radiotherapy and chemotherapy as treatment options for late recurrence (Caruso et al., 2021). However, the general state of advanced patients is poor, whether they can tolerate the operation after neoadjuvant chemotherapy, whether the operation area can achieve tumor-free status, and whether the treatment will prolong the survival time of patients still need to be further discussed. At present, it is reported that angiogenesis inhibitors, cell signaling pathway inhibitors, immunotherapy, and apoptosis promoters can be used to detect lung SCNEC (Abidin et al., 2010). Therefore, we need to find gene changes suitable for targeted therapy, so as to complete the application of these treatments in cervical SCNEC. Salani et al. treated recurrent small cell neural cell carcinoma with trametinib. The patient was accompanied by a-kras mutation and achieved complete remission after 3 cycles of treatment (Salani et al., 2011). A recent study using next-generation sequencing technology to test cancer-related genes has shown that several targeted mutations (KRAS, PIK3CA, IRS2, Sox2, and HRR genes) may potentially become effective targeted treatment sites for patients (Pei et al., 2021). Eskander et al. analyzed the whole genome of 97 patients with cervical small cell carcinoma. They suggested that drugs targeting PIK3CA and other genes may become a potential treatment (Eskander et al., 2020). Furthermore, many previous studies have shown that PD-L1 is a notable manifestation in cervical carcinoma (Reddy et al., 2017; Chinn et al., 2019), and some scholars have also confirmed that treatment of immune check-point is working in patients with PD-L1 positive and MMRS deficiency (Ji et al., 2021).

## Conclusions

The characteristics of cervical SCNEC are high malignancy, high invasiveness, and high mortality. It is interrelated with high risk HPV while HPV 18 carries a higher risk of cevical SCNEC. We found that multimodal treatment can indeed improve tumor-free survival (DFS), but the prognosis is poor. We commonly used neuroendocrine staining agents are CGA, SYN, CD 56, P16, Ki67, and neuron-specific NSE. For the early stages, our treatment principles refer to the guidelines of SGO, international gynecological cancer Cooperation (GCIG), and NCCN. We suggest a combination of surgery, radiotherapy, and chemotherapy. In order to better prolong tumor-free survival of patients, we need to find gene changes suitable for targeted therapy, so as to complete the application of these treatments in cervical SCNEC. Further studies are needed to explore more effective therapy for cervical SCNEC.

## Data Availability Statement

The raw data supporting the conclusions of this article will be made available by the authors, without undue reservation.

## Ethics Statement

Written informed consent from the patients/participants was not required to participate in this study in accordance with the national legislation and the institutional requirements.

## Author Contributions

JW dealt with the case and drafted the manuscript. JL assisted in the collection and statistics of case data, and carried out all the documentary and article work out. All authors read and approved the final manuscript.

## Conflict of Interest

The authors declare that the research was conducted in the absence of any commercial or financial relationships that could be construed as a potential conflict of interest.

## Publisher’s Note

All claims expressed in this article are solely those of the authors and do not necessarily represent those of their affiliated organizations, or those of the publisher, the editors and the reviewers. Any product that may be evaluated in this article, or claim that may be made by its manufacturer, is not guaranteed or endorsed by the publisher.
